# Cytokine Secretion and Pyroptosis of Thyroid Follicular Cells Mediated by Enhanced NLRP3, NLRP1, NLRC4, and AIM2 Inflammasomes Are Associated With Autoimmune Thyroiditis

**DOI:** 10.3389/fimmu.2018.01197

**Published:** 2018-06-04

**Authors:** Qingling Guo, Ying Wu, Yuanyuan Hou, Yongping Liu, Tingting Liu, Hao Zhang, Chenling Fan, Haixia Guan, Yushu Li, Zhongyan Shan, Weiping Teng

**Affiliations:** ^1^Department of Endocrinology and Metabolism, Institute of Endocrinology, Liaoning Provincial Key Laboratory of Endocrine Diseases, The First Affiliated Hospital of China Medical University, Shenyang, China; ^2^Department of Thyroid Surgery, The First Affiliated Hospital of China Medical University, Shenyang, China

**Keywords:** autoimmune thyroiditis, inflammasome, pyroptosis, interleukin-18 (IL-18), absent in melanoma 2

## Abstract

**Background:**

Inflammasomes, which mediate maturation of interleukin-1β (IL-β) and interleukin-18 (IL-18) and lead to pyroptosis, have been linked to various autoimmune disorders. This study investigated whether they are involved in the pathogenesis of autoimmune thyroiditis (AIT).

**Methods:**

We collected thyroid tissues from 50 patients with AIT and 50 sex- and age-matched controls. Serum levels of free T3, free T4, thyrotropin, thyroid peroxidase antibody (TPOAb), and thyroglobulin antibody (TgAb) were measured by electrochemiluminescent immunoassays. Expression of several inflammasome components, the NOD-like receptor (NLR) family pyrin domain containing 1 (NLRP1), NLRP3, CARD-domain containing 4 (NLRC4), absent in melanoma 2 (AIM2), the apoptosis-associated speck-like protein that contains a caspase recruitment domain (ASC), caspase-1, IL-1β, and IL-18 was determined by real-time PCR and western blot. Immunohistochemistry was used to localize the expression of NLRP1, NLRP3, NLRC4, and AIM2. The Nthy-ori 3-1 thyroid cell line was stimulated with tumor necrosis factor-α (TNF-α), interferon-γ (IFN-γ), interleukin-17A, interleukin-6, and poly(dA:dT). The levels of IL-18 and IL-1β in the cell supernatant were measured by enzyme-linked immunosorbent assay, and lactate dehydrogenase was quantified by absorptiometry. ASC specks were examined by confocal immunofluorescence microscopic analysis. Cell death was examined by flow cytometry, and the N-terminal domain of gasdermin D was detected by western blot analysis.

**Results:**

Expression of NLRP1, NLRP3, NLRC4, AIM2, ASC, caspase-1, pro IL-1β, pro IL-18, mRNA, and protein was significantly increased in thyroid tissues from patients with AIT, and enhanced posttranslational maturation of caspase-1, IL-18 and IL-1β was also observed. Expression of NLRP1, NLRP3, NLRC4, and AIM2 was localized mainly in thyroid follicular cells adjacent to areas of lymphatic infiltration. The thyroid mRNA level of NLRP1 and ASC was correlated to the serum TPOAb and TgAb levels in the AIT group. TNF-α and IFN-γ had a priming effect on the expression of multiple inflammasome components in thyroid cells. IFN-γ was found to strengthen poly(dA:dT)-induced cell pyroptosis and bioactive IL-18 release.

**Conclusion:**

Our work has demonstrated for the first time that multiple inflammasomes are associated with AIT pathogenesis. The identified NLRP3, NLRP1, NLRC4, AIM2 inflammasomes and their downstream cytokines may represent potential therapeutic targets and biomarkers of AIT.

## Introduction

Autoimmune thyroiditis (AIT), a typical organ-specific autoimmune disorder, is the main cause of hypothyroidism and affects 10% of the population in China (1, 2). AIT is characterized by the presence of thyroid-specific autoantibodies in the serum, massive infiltration of lymphocytic cells, and destruction of the follicular structure within the thyroid (1, 3). Genetic susceptibility, together with environmental factors, contributes to the breakdown of immune tolerance, but the specific molecular events are not clear yet (4). With regard to tissue-specific autoimmunity, apart from aberrant functioning of the immune system, abnormality of thyroid follicular cells (TFCs) is an important contributing factor (5). In fact, TFCs have been reported to express toll-like receptors in response to various pathogen-associated molecular patterns (PAMPs) and endogenous damage-associated molecular patterns (DAMPs), and to induce activation of the innate immune system (5–7). This leads to the chemotaxis of self-reactive lymphocytes to the thyroid, where these lymphocytes produce multiple pro-inflammatory cytokines, such as interleukin-1β (IL-1β), interferon-γ (IFN-γ), and tumor necrosis factor-α (TNF-α), which cause injury or apoptosis in TFCs and contribute to the pathogenesis of AIT (8–10). In addition to this, previous studies have demonstrated the important role of IL-1β in AIT development, *via* induction of the expression of Fas/FasL and intercellular adhesion molecule-1 on TFCs and disturbance of the integrity of the thyroid epithelium (8, 11, 12). Moreover, high levels of IL-18, a constitutively expressed cytokine, evidently potentiates the production of IFN-γ from Th1 cells and natural killer cells (13), as observed in TFCs from patients with AIT (9, 14). Thus, the findings so far indicate that TFC itself and various pro-inflammatory cytokines play an important role in the pathogenesis of AIT.

Inflammasomes are intracellular multi-protein plats composed of NOD-like receptors (NLRs)/AIM-like receptors, the apoptosis-associated speck-like protein that contains caspase activation recruitment domain (ASC) and the inflammatory effector pro caspase-1 (15). Activation of inflammasomes leads to proteolytically activation of the cysteine protease caspase-1 and produce a tetramer of its two active subunits—p20 and p10. caspase-1 p20 mediates maturation of pro IL-1β and pro IL-18; this is followed by gasdermin D-mediated programmed inflammatory cell death or pyroptosis, and subsequently, the release of active cytokines. Indeed, the level of caspase-1 p20 has been considered as an indicator of inflammasome activity (15, 16). The NLR family, pyrin domain containing 3 (NLRP3), pyrin domain containing 1 (NLRP1), and caspase activation recruitment domain containing 4 (NLRC4) are the most studied intracellular pattern recognition receptors; they are known for their ability to assemble canonical inflammasomes in response to specific PAMPs and DAMPs (17). On the other hand, the HIN-200 family member absent in melanoma 2 (AIM2) is a direct receptor for cytosolic double-strand DNA and the main component of the AIM2 inflammasome (18). Inflammasomes have been identified primarily in peripheral monocytes, but recent studies have demonstrated their expression in tissue cells, such as islet-β cells (19), neurons (20), and keratinocytes, too (21). Aberrant expression or dysregulated function of inflammasomes has been linked to autoimmunity and organ damage (22, 23), and also various diseases and their progression. For example, Marie and colleagues reported increased expression of NLRP3 at baseline and enhanced secretion of IL-1β after stimulation of whole blood cells from patients with rheumatoid arthritis (24). In another study, the expression levels of NLRP1 and IL-1β in perilesional vitiligo/non-segmental vitiligo skin were found to be significantly associated with disease progression (25). Based on these findings, it is possible that aberrant activity or expression of inflammasomes is also involved in the pathogenesis of AIT. However, no study so far has explored the role of inflammasomes in AIT.

The aim of the current study was to investigate the expression signature of several classical inflammasomes—the NLRP1, NLRP3, NLRC4, and AIM2 inflammasomes—in AIT patients and controls. In addition, a human thyroid follicular epithelial cell line (Nthy-ori 3-1) was used to explore the potential pathogenic roles of inflammasome activation. Our work is the first to demonstrate enhanced expression and activity of multiple inflammasomes in thyroid tissues from AIT patients, and also the role of the AIM2 inflammasome in the pyroptosis of TFCs and release of mature IL-18.

## Materials and Methods

### Subjects and Samples

This study was conducted at the First Hospital of China Medical University from December 2015 to August 2017. We recruited 100 patients with benign thyroid nodules who were scheduled for thyroidectomy. Thyroid specimens adjacent to the nodules (at least 2 cm away from the nodule) were collected. Fifty patients with pathologically diagnosed AIT on intraoperative biopsy and positive for serum thyroid peroxidase antibody (TPOAb) or thyroglobulin antibody (TgAb) (TPOAb > 5.61 IU/ml, TgAb > 4.11 IU/ml; standard values obtained from Abbott) were enrolled as the AIT group. Age- and sex-matched subjects who were negative for autoantibodies were enrolled as the control (CON) group. All subjects included in both AIT and CON groups were euthyroid, and none of them with l-thyroxine substitution. The following exclusion criteria were applied: (1) existence of other autoimmune disorders and chronic inflammatory diseases, such as systemic lupus erythematosus, rheumatic disease, Sjogren disease, inflammatory bowel disease, psoriasis, vitiligo, diabetes mellitus, and gout; (2) existence of acute or chronic infections, such as viral hepatitis and HIV infection; (3) existence of pregnancy, malignancy, and current medication use. All research procedures were approved by the Medical Ethics Committee of the First Hospital of China Medical University. Both informed and written consent were obtained from all participants.

A part of the thyroid tissue specimens was stored at −80°C for total RNA and protein extraction, and a part of it was immersed in 4% paraformaldehyde to make paraffin blocks. Serum samples were collected before surgery and analyzed as soon as possible.

### Cell Culture and *In Vitro* Stimulation

The Nthy-roi 3-1 cell line was obtained from Prof. Hao Zhang, Department of Thyroid Surgery, the First Hospital of China Medical University. Cells were cultured in RPMI 1640 medium containing 2 mM glutamine (#31800; Solarbio Life Science, Beijing, China) supplemented with 10% fetal bovine serum. Cells were treated with recombinant human IFN-γ (#285-IF; R&D, USA) at concentrations of 250, 500, and 1,000 IU/ml; TNF-α (#210-TA; R&D, USA) at concentrations of 125, 250, and 500 IU/ml; IL-17A (#061184; Peprotech, USA) at concentrations of 0.1, 1, and 10 ng/ml; and IL-6 (NBP2-34901; Novus Biologicals, USA) at concentrations of 0.1, 1, and 10 ng/ml for 24 h and placed in Trizol reagent for total RNA extraction. For *in vitro* AIM2 inflammasome activation, cells were incubated with 1 µg/ml poly(dA:dT) for 12 h after priming with 250 IU/ml IFN-γ for 24 h and replacement with new medium; moreover, one group of cells was treated only with poly(dA:dT) and one group was treated only with IFN-γ.

### Biochemical Measurements

Serum levels of free T3, free T4, thyrotropin (TSH), TPOAb, and TgAb were measured by electrochemiluminescent immunoassays on Architect i2000SR (Abbott Laboratories, Chicago, IL, USA). Reference ranges were obtained from the manufacturer.

### Total RNA Extraction and Real-Time PCR (RT-PCR)

Total RNA was extracted from thyroid tissues and cultured thyroid cells with Trizol reagent (#9109; TaKaRa, Japan), and RNA concentration was determined on a Nanodrop 2000C spectrophotometer (Nano Drop Technologies, USA). Samples with an OD260/OD280 ratio ranging between 1.8 and 2.0 were used for experiments. Reverse transcription reactions were carried out on a VeritiTM 96-well Thermal Cycler (AB Applied Biosystems, Singapore) with 1,000 ng RNA per sample and a 5 × PrimeScript RT reagent kit (#036A; TaKaRa, Japan). PCR amplifications were performed on a LightCycler 480 Real-Time PCR System (Roche, Switzerland) using a SYBR Premix Ex Taq™ II kit (#820A; TaKaRa, Japan), and GAPDH was used as an internal reference. Primers were synthesized by TaKaRa Biotech (specific sequences are shown in Table 1). Each sample was analyzed in duplicate. A melting curve was generated during amplification to verify the absence of primer dimers or incorrectly paired products. Relative mRNA expression levels of the target genes were calculated using the 2^−ΔCp^ method after they were corrected based on GAPDH expression.

**Table 1:** Primer sequences for real-time PCR.

Gene	Sequences (5′ to 3′)
NLRP1	F: CCACAACCCTCTGTCTACATTAC; R: GCCCCATCTAACCCATGCTTC
NLRP3	F: GATCTTCGCTGCGATCAACA; R: GGGATTCGAAACACGTGCATTA
NLRC4	F: CCAGTCCCCTCACCATAGAAG; R: ACCCAAGCTGTCAGTCAGACC
AIM2	F: CTGCAGTGATGAAGACCATTCGTA; R: GGTGCAGCACGTTGCTTTG
ASC	F: AACCCAAGCAAGATGCGGAAG; R: TTAGGGCCTGGAGGAGCAAG
CASP1	F: GCCTGTTCCTGTGATGTGGAG; R: TGCCCACAGACATTCATACAGTTTC
IL-1B	F: CCAGGGACAGGATATGGAGCA; R: TTCAACACGCAGGACAGGTACAG
IL-18	F: CTGCCACCTGCTGCAGTCTA; R: TCTACTGGTTCAGCAGCCATCTTTA
GAPDH	F: GCACCGTCAAGGCTGAGAAC; R: TGGTGAAGACGCCAGTGGA

*F, forward; R, reverse*.

### Total Protein Extraction and Western Blot Analysis

Total protein was extracted from thyroid tissues and cultured thyroid cells with the Minute™ Total Protein Extraction Kit for Animal Cultured Cells and Tissues (#SD-001/SN-002; Invent Biotechnologies Inc., USA). Protein concentration was estimated using the BCA Protein Concentration Assay Kit (#P0012S; Beyotime, China) and adjusted to 4 µg/µl. Samples with equal concentrations of proteins were boiled at 100°C for 6 min, and 15-µl aliquots of each sample were loaded onto the 10% sodium dodecyl sulfate-acrylamide gels. Protein molecules were separated at a constant voltage of 120 V and then transferred onto nylon membranes. After blocking with 5% skimmed milk, membranes were incubated overnight at 4°C with rabbit antibodies against human NLRP3 (#15101 at a 1:1,000 dilution; Cell Signal Technology, USA), AIM2 (#12948 at a 1:1,000 dilution; Cell Signal Technology, USA), NLRP1 (#12256-1-AP at a 1:1,000 dilution; Proteintech Group Inc., USA), NLRC4 (#A7382 at a 1:1,000 dilution; AB Clonal Inc., USA), ASC (#ab155970 at a 1:1,000 dilution; Abcam, UK), pro caspase-1 (#2225 at a 1:1,000 dilution; Cell Signal Technology, USA), cleaved caspase-1 (#4199 at a 1:1,000 dilution; Cell Signal Technology, USA), pro IL-1β (#ab156791 at a 1:2,000 dilution, Abcam, UK), cleaved IL-1β (#83186 at a 1:1,000 dilution; Cell Signal Technology, USA), pro IL-18 (#10663-1-AP at a 1:1,000 dilution; Proteintech Group Inc., USA), cleaved IL-18 (# sc-7954 at a 1:200 dilution; Santa Cruz, CA, USA), and Gasdermin D (#20770 at a 1:1,000 dilution; Proteintech Group Inc., USA). Rabbit anti-GAPDH antibody (sc-25778 at a 1:1,000 dilution; Santa Cruz, CA, USA) was used as an internal reference. Membranes were washed with Tris-buffered saline containing 0.1% Tween-20 for 5 min three times and incubated with peroxidase-conjugated goat anti-rabbit IgG (#7074 at a 1:10,000 dilution; Cell Signaling Technology, USA) for 1 h at room temperature. Membranes were reacted with an enhanced chemiluminescence solution (#P0018; Beyotime, China) after washing, and were exposed to film for imaging. Protein bands were quantified with the Image J 2.0 software. The ratio of the intensity of target protein bands to GAPDH band intensity was calculated.

### Immunohistochemistry

A series of paraffin sections (4-µm thickness) of thyroid tissue were made on a microtome (Leica RM 2135, Germany), and immunohistochemically analyzed with an EliVision™ super kit (#9923; MXB Biotechnologies, Fuzhou, China). In brief, tissue sections were dewaxed, rehydrated, and incubated with 0.01 M citrate buffer for 10 min in a microwave oven for antigen repair. Then, the sections were incubated with 3% hydrogen peroxide to block endogenous peroxidase activity. After washing with phosphate-buffered saline (PBS), the sections were blocked with 5% bovine serum albumin for 1 h at room temperature. The sections were then incubated with rabbit antibodies against human NLRP3 (#19771-1-AP at a 1:100 dilution; Proteintech Group Inc., USA), NLRP1 (#12256-1-AP at a 1:100 dilution; Proteintech Group Inc., USA), NLRC4 (#A7382 at a 1:50 dilution; AB Clonal Inc., USA) and AIM2 (#20590-1-AP at a 1:100 dilution; Proteintech Group Inc., USA) in a humidity box at 4°C overnight. After the sections were rinsed with PBS, horseradish peroxidase-conjugated anti-rabbit IgG antibody was added to the tissue slices and they were incubated at 37°C for 30 min. Finally, the reaction was visualized in brown color under a light microscope after incubation with diamino-3,3′-benzidine tetrahydrochloride for 3–5 min. The tissues were counterstained with hematoxylin. Negative controls were not treated with the primary antibody. Expression level of the proteins was evaluated using the Image-Pro Plus 5.1 software.

### Immunofluorescence and Confocal Microscopy

Thyroid cells were grown on chamber slides, stimulated with 250 IU/ml IFN-γ for 24 h and then incubated with 1 µg/ml poly(dA:dT) for 12 h after they had adhered. Cells were fixed in 4% paraformaldehyde after washing three times with cold PBS. The cell slides were blocked with 5% bovine serum albumin for 30 min at room temperature, and the cell membranes were penetrated with the addition of 0.3% Triton X-100 in blocking solution. Then, the cells were incubated overnight at 4°C with rabbit antibody against human AIM2 (#20590-1-AP at a 1:100 dilution; Proteintech Group Inc., USA) and mouse monoclonal antibody against human ASC (sc-514414 at a 1:100 dilution; Santa Cruz, CA, USA). Afterward, the cells were incubated with FITC-conjugated anti-mouse (IF0091 at a 1:150 dilution; DingGuo Biotech, Beijing, China) and Cy3-conjugated anti-rabbit IgG (#AS007 at a 1:200 dilution; AB Clonal Inc., USA) after rinsing with PBS containing 0.1% Tween-20 three times. Ultimately, the cell slides were mounted in an anti-fade reagent (#S2100; Solarbio Life Science, Beijing, China) after staining with 4, 6-diamidino-2-phenylindole (#C1005; Beyotime, China) and observed under a confocal laser-scanning microscope (Leica Microsystem CMS GmbH, Germany).

### Enzyme-Linked Immunosorbent Assay (ELISA)

The concentration of IL-18 and IL-1β in cell supernatants was determined by ELISA using commercial quantitative kits according to the manufacturer’s instructions (#VAL101, Valukine™ ELISA Kit for human IL-1β; R&D, USA) (#BMS267-2, Human IL-1 8 Platinum ELISA; eBioscience, USA). All samples were analyzed in duplicate. Standard curves were generated during assays by plotting absorbance value against the gradient concentration of the standards provided with the kits. Both positive and blank controls were analyzed simultaneously on the same plate.

### Lactate Dehydrogenase (LDH) Analysis

Lactate dehydrogenase concentration in the supernatant was measured using the CytoTox 96 Non-Radioactive Cytotoxicity Assay kit (#1780; Promega, USA) according to the manufacturer’s instructions. Thyroid cells were treated as indicated. No-cell controls were set up to serve as the negative control to determine the culture medium background. Untreated cells served as a vehicle control. The lysis solution given in the kit was used to generate a maximum LDH release control. Briefly, 50-µl aliquots from the test and control wells were transferred to a fresh 96-well flat clear bottom plate, 50 µl of the CytoTox 96^®^ reagent was added and the wells were incubated for 30 min at room temperature. Eventually, 1 h after the stop solution was added, absorbance was measured at 490 nm. The average values of the culture medium background were subtracted from the values of the experimental wells. The percentage of cell death was calculated using the following formula:

$$\text{Percent cell death }(\text{\%})=\text{100}\times \frac{{\text{Experimental LDH Release (OD}}_{490})}{{\text{Maximum LDH Release (OD}}_{490})}.$$

### Flow Cytometry

Annexin V-propidium iodide (PI) staining was carried out with an FITC-Annexin V Apoptosis Detection Kit with PI (#640914; BioLegend, San Diego, CA, USA). Cells were detached and washed three times with cold BioLegend cell staining buffer, and then resuspended in Annexin V binding buffer at a concentration of 5 × 10^6^/ml. Following this, 100 µl of cell suspension was transferred to a flow tube, and the cells were incubated with FITC-Annexin V and PI for 15 min at room temperature in the dark. Eventually, 400 µl of Annexin V binding buffer was added to each tube, and the samples were analyzed on a Becton Dickinson FACS instrument (BD, USA). An experienced technician in our laboratory was responsible for the machine settings. FlowJo 7.6 software was used for data analysis.

### Statistical Analysis

Statistical analyses were performed with the SPSS 21.0 software. Comparison of normally distributed quantitative variables between the AIT and CON group was carried out using Student’s *t*-tests, while comparison of non-normally distributed parameters (TPOAb and TgAb) was conducted using non-parametric Mann–Whitney tests. Categorical variables (sex and cigarette consumption) were presented as frequencies, and chi-square tests were used to compare the AIT and CON groups. Bivariate correlation analysis was performed using the Pearson or Spearman rank correlation method. Statistical analyses of the different cell stimulation groups were conducted using one-way ANOVA followed by Bonferroni correction for paired comparisons. All calculated *P* values were two-sided, and *P* values < 0.05 were considered to indicate statistical significance.

## Results

### Increased mRNA and Protein Expression of Inflammasome Components in Thyroid Tissues From AIT Patients

**Figure 1:**
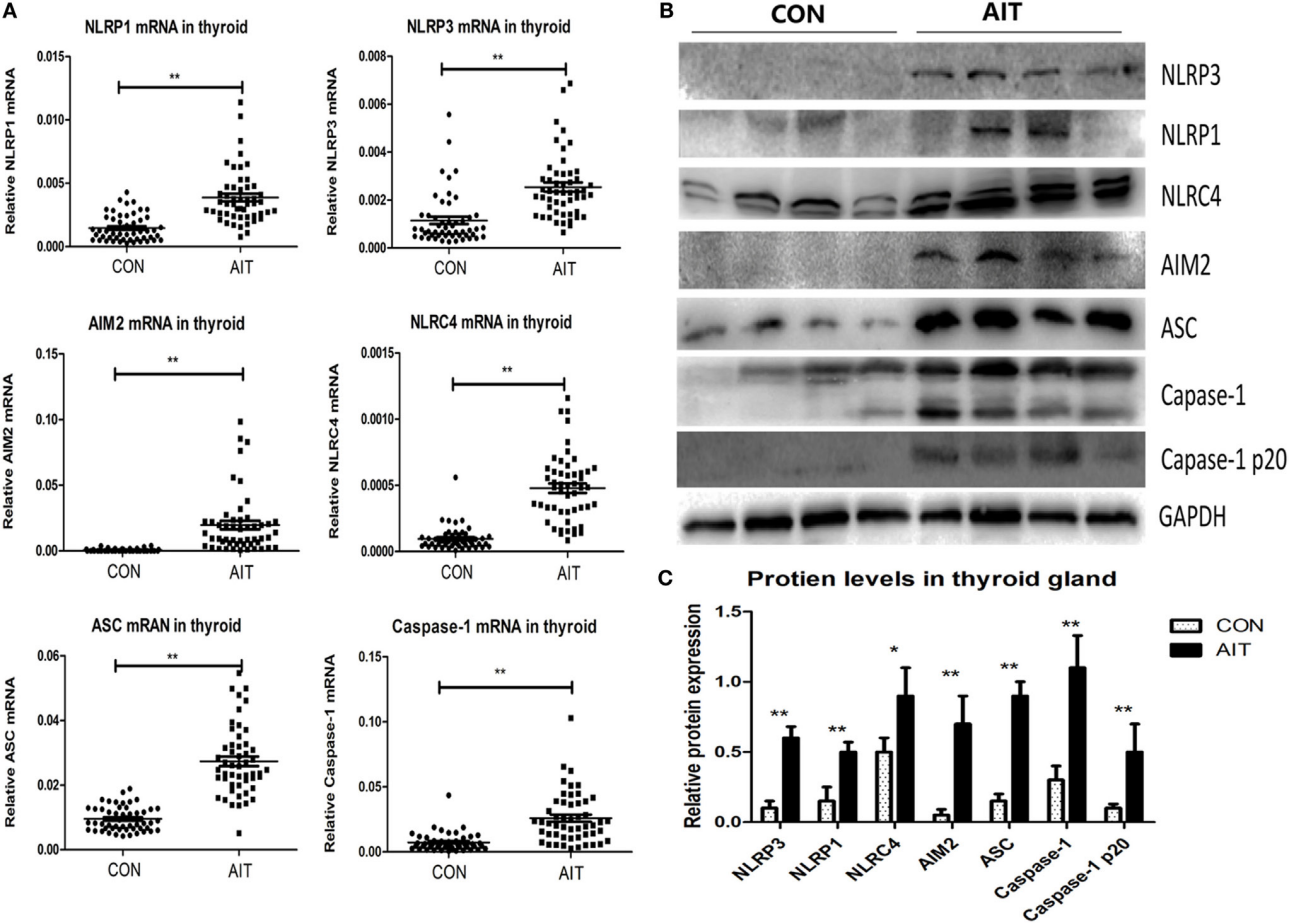
Increased mRNA and protein expression of inflammasome components in thyroid tissues from AIT patients. **(A)** The mRNA expression levels of NLRP1, NLRP3, NLRC4, absent in melanoma 2 (AIM2), ASC, and caspase-1 in thyroid tissues from AIT patients and controls (*N* = 50 in each group). Representative stripe images **(B)** and corresponding quantitative analysis **(C)** of the NLRP1, NLRP3, NLRC4, AIM2, ASC, caspase-1 and caspase-1 p20 protein levels in thyroid tissues from AIT patients and controls (*N* = 20 in each group). The relative mRNA and protein expression levels were corrected to GAPDH expression. The columns represent the mean ± SD values. Student’s *t*-test or Mann–Whitney *U*-test was used for comparison between the AIT and control groups. *P < 0.05, **P < 0.01. AIT, autoimmune thyroiditis; CON, controls.

The demographic and clinical characteristics of all the subjects are summarized in Table 2. There were no statistically significant differences in age, sex ratio, body mass index (BMI), and cigarette consumption between the two groups, which indicates a comparable baseline. The subjects included in both groups were all euthyroid, but the serum thyrotropin level in the AIT patients was higher than that in the controls (2.36 ± 1.43 vs. 1.68 ± 0.89, *P* = 0.006). As shown in Figure 1A, mRNA expression of NLRP1, NLRP3, NLRC4, and AIM2 in the thyroid of AIT patients was significantly higher than that of the controls (*P* < 0.01 for all). As common components of several inflammasomes, ASC and caspase-1 mRNA expression was also markedly elevated in AIT patients (*P* < 0.01 for both). Figures 1B,C show that the NLRP1, NLRP3, NLRC4, AIM2, ASC, and caspase-1 protein levels in thyroid tissues from patients with AIT were evidently higher than those in tissues from the controls (*P* < 0.05 for all the proteins).

**Table 2:** Demographic and clinical characteristics of the AIT patients and controls.

	CON	AIT	*P*-value
Age (years)^a^	46.8 ± 10.8	44.4 ± 11.9	0.297
Sex (F/M)^c^	2/48	3/47	0.64
BMI (kg/m^2^)^a^	22.38 ± 2.32	22.41 ± 2.37	0.944
Smoking^c^	3/47	5/45	0.724
FT3 (pmol/l)^a^	4.56 ± 0.48	4.47 ± 0.47	0.378
FT4 (pmol/l)^a^	13.3 ± 1.34	13.2 ± 1.70	0.870
TSH (mIU/l)^a^	1.68 ± 0.89	2.36 ± 1.43	0.006**
TPOAb (IU/ml)^b^	0.19 (0.08–0.42)	101.59 (10.16–457.16)	<0.001**
TgAb (IU/ml)^b^	1.06 (0.77–1.52)	66.91 (16.51–264.65)	<0.001**

*^a^Variables are shown as the mean ± SD values. Comparisons between groups were conducted using Student’s t-test*.

*^b^Variables are shown as the median values with their interquartile range. Comparisons between groups were conducted using the Mann–Whitney U-test*.

^c^Categorical variables are shown as frequencies, and the chi-square test was applied for comparisons between groups. **P < 0.01, AIT vs. control group.

*The normal reference ranges were obtained from the manufacturer (Abbott): free T3 (FT3): 2.63–5.70 pmol/l; free T4 (FT4): 9.01–19.05 pmol/l; thyrotropin (TSH): 0.35–4.94 mIU/l; thyroid peroxidase antibody (TPOAb): 0.00–5.61 IU/ml; thyroglobulin antibody (TgAb): 0.00–4.11 IU/ml*.

*F, female; M, male; BMI, body mass index; AIT, autoimmune thyroiditis; CON, controls*.

### Enhanced Activation of Inflammasomes in Thyroid Tissues From AIT Patients

**Figure 2:**
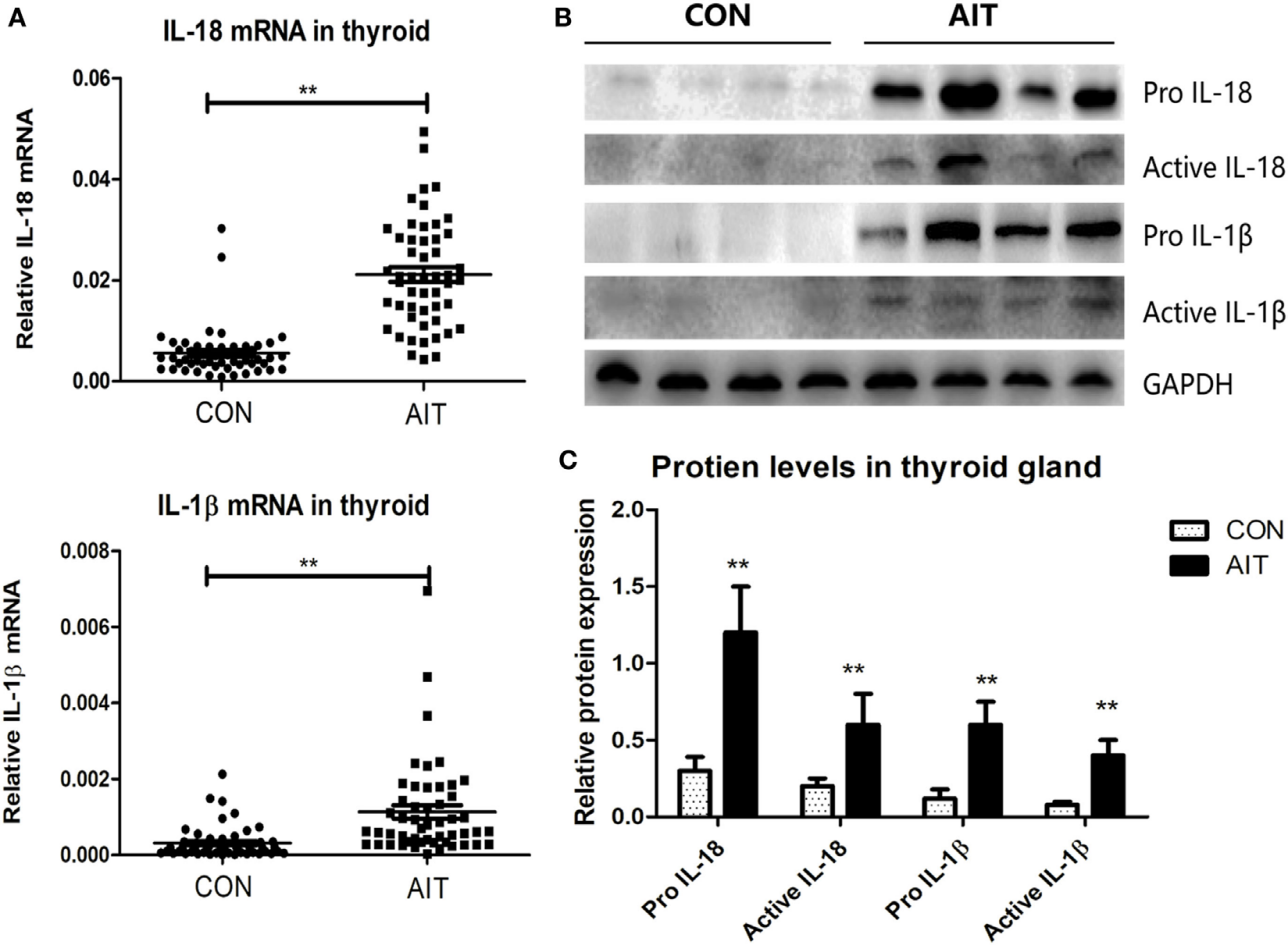
Increased expression and maturation of IL-18 and IL-1β in thyroid tissues from AIT patients. **(A)** The mRNA expression levels of IL-18 and IL-1β in thyroid tissues from AIT patients and controls (*N* = 50 in each group). Representative stripe images **(B)** and corresponding quantitative analysis **(C)** of pro IL-18, active IL-18, pro IL-1β, and active IL-1β protein levels in thyroid tissues from AIT patients and controls (*N* = 20 in each group). The relative mRNA and protein expression levels were corrected to GAPDH expression. The columns represent the mean ± SD values. Student’s *t*-test or Mann–Whitney *U*-test was used for comparison between the AIT and control groups. *P < 0.05, **P < 0.01. AIT, autoimmune thyroiditis; CON, controls.

As shown in Figures 1B,C, elevated p20 protein level (the active form of caspase-1) (26) was observed in the AIT group, but it was scarcely expressed in the control group (*P* < 0.01). We observed significantly higher mRNA and protein expression of pro IL-1β and pro IL-18 in thyroid tissues from AIT patients (*P* < 0.01 for the mRNA expression of both pro IL-1β and pro IL-18; *P* < 0.01 for the protein expression of both pro IL-1β and pro IL-18). More notably, increased levels of active IL-1β and active IL-18 (*P* < 0.01 for both) were detected in AIT patients, but they were almost absent in the controls (Figure 2). These findings may indicate enhanced inflammasome activation in the thyroid of AIT patients.

### Localization of Enhanced Expression of NLRP1, NLRP3, NLRC4, and AIM2 in the Cytoplasm of TFCs

**Figure 3:**
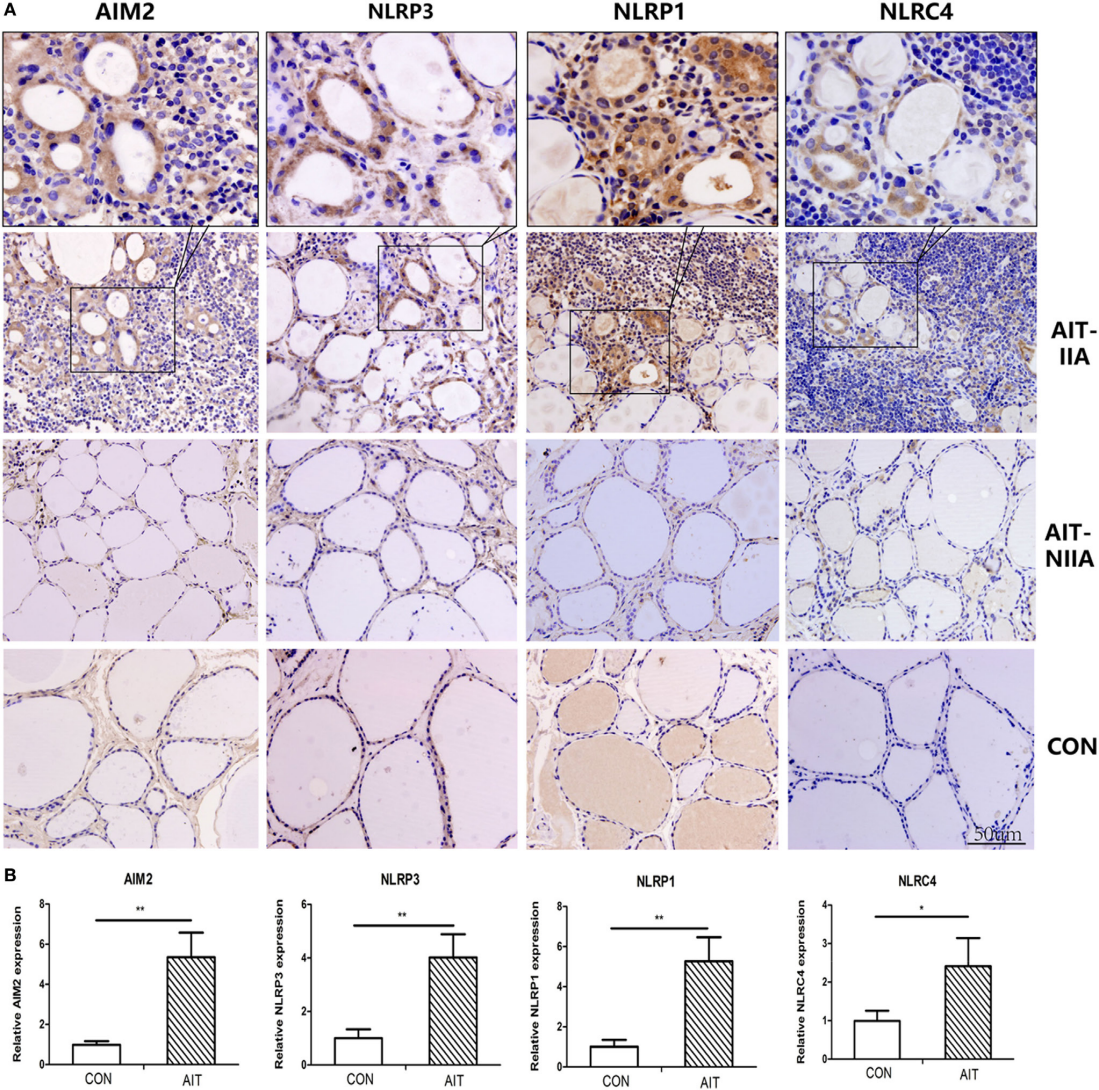
Localization of enhanced expression of NLRP1, NLRP3, NLRC4, and absent in melanoma 2 (AIM2) in thyroid cells in areas of lymphatic infiltration. **(A)** Representative immunohistochemical staining images of AIM2, NLRP3, NLRP1, and NLRC4 in thyroid tissue sections from AIT patients and controls (400× magnification, light microscope). **(B)** Quantified expression levels of AIM2, NLRP3, NLRP1, and NLRC4 in control and AIT tissues (*N* = 20 in each group). The columns represent the mean ± SD values. Three views were randomly selected in each subject. Statistical analysis was carried out using Student’s *t*-test. *P < 0.05, **P < 0.01. AIT, autoimmune thyroiditis; CON, controls; IIA, inflammatory infiltration area; NIIA, non-inflammatory infiltration area.

As shown in Figure 3A, expression of NLRP1, NLRP3, NLRC4, and AIM2 was mainly localized in the cytoplasm of the TFCs. Swollen follicular epithelial cells in areas containing infiltrating lymphocytes demonstrated significantly intense expression of these proteins, while TFCs expressing these proteins were only rarely observed in thyroid tissue from AIT patients without lymphocyte infiltration. In contrast, expression of these proteins was low in the control thyroid tissue (Figures 3A,B).

### Correlation Between mRNA Levels of NLRP1, NLRP3, NLRC4, AIM2, ASC, caspase-1, IL-1β, and IL-18 in Thyroid Tissues and Serum Autoantibodies

**Figure 4:**
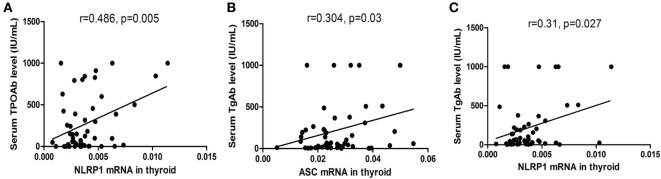
Correlation between the mRNA levels of inflammasome components in thyroid tissues and autoantibody levels in the serum in autoimmune thyroiditis (AIT) patients. Correlation between the NLRP1/ASC mRNA levels in thyroid tissue and serum thyroid peroxidase antibody (TPOAb)/thyroglobulin antibody (TgAb) levels in the AIT group **(A–C)**. *N* = 50 in each group. A bivariate correlation analysis was performed using the Spearman rank test. *R* represents the Spearman correlation coefficient.

As shown in Figure 4A, we observed a positive correlation between the NLRP1 mRNA level and serum TPOAb level (*r* = 0.486, *P* = 0.005, *n* = 50) in patients with AIT. In addition, the serum TgAb levels were positively correlated with the mRNA levels of NLRP1 (*r* = 0.310, *P* = 0.027, *n* = 50) and ASC (*r* = 0.304, *P* = 0.03, *n* = 50) in thyroid samples from AIT patients (Figures 4B,C).

Correlations between the mRNA levels of NLRP3, NLRP1, NLRC4, AIM2, ASC, caspase-1, IL-1β, and IL-18 are summarized in Tables 3 and 4. A general positive correlation was observed both in the AIT and control group.

**Table 3:** Correlation between NLRP3, NLRP1, NLRC4, absent in melanoma 2 (AIM2), ASC, caspase-1, IL-1β, and IL-18 expression in the autoimmune thyroiditis group.

		NLRP3	NLRP1	NLRC4	AIM2	ASC	Caspase-1	IL-1β	IL-18
NLRP3	*r*	1	0.466**	0.660**	0.385**	0.207	0.367**	0.288*	0.687**
	*P*		<0.001	<0.001	0.005	0.144	0.008	0.040	<0.001

NLRP1	*r*	0.466**	1	0.366**	0.121	0.492**	0.300*	0.023	0.489**
	*P*	0.001		0.008	0.399	<0.001	0.032	0.872	<0.001

NLRC4	*r*	0.660**	0.366**	1	0.656**	0.476**	0.777**	0.307*	0.862**
	*P*	<0.001	0.008		<0.001	<0.001	<0.001	0.028	<0.001

AIM2	*r*	0.385**	0.121	0.656**	1	0.370**	0.698**	0.254	0.656**
	*P*	0.005	0.399	<0.001		0.008	<0.001	0.072	<0.001

ASC	*r*	0.207	0.492**	0.476**	0.370**	1	0.596**	0.086	0.576**
	*P*	0.144	<0.001	<0.001	0.008		<0.001	0.548	<0.001

Caspase-1	*r*	0.367**	0.300*	0.777**	0.698**	0.596**	1	0.217	0.839**
	*P*	0.008	0.032	<0.001	<0.001	<0.001		0.126	<0.001

IL-1β	*r*	0.288*	0.023	0.307*	0.254	0.086	0.217	1	0.311*
	*P*	0.040	0.872	0.028	0.072	0.548	0.126		0.026

IL-18	*r*	0.687**	0.489**	0.862**	0.656**	0.576**	0.839**	0.311*	1
	*P*	<0.001	<0.001	<0.001	<0.001	<0.001	<0.001	0.026	

*Bivariate correlation analysis was carried out using the Pearson correlation method*.

*Statistically significant at P < 0.05.

**Statistically significant at P < 0.01.

**Table 4:** Correlation between NLRP3, NLRP1, NLRC4, absent in melanoma 2 (AIM2), ASC, caspase-1, IL-1β, and IL-18 expression in the CON group.

		NLRP3	NLRP1	NLRC4	AIM2	ASC	Caspase-1	IL-1β	IL-18
NLRP3	*r*	1	0.456**	0.450**	0.338*	0.270	0.112	0.533**	0.290*
	*P*		0.001	0.001	0.023	0.053	0.427	<0.001	0.037

NLRP1	*r*	0.456**	1	0.230	0.085	0.168	0.066	0.213	0.152
	*P*	0.001		0.108	0.578	0.235	0.644	0.129	0.282

NLRC4	*r*	0.450**	0.230	1	0.758**	0.610**	0.428**	0.231	0.743**
	*P*	0.001	0.108		<0.001	<0.001	0.002	0.106	<0.001

AIM2	*r*	0.338*	0.085	0.758**	1	0.675**	0.748**	0.282	0.812**
	*P*	0.023	0.578	<0.001		<0.001	<0.001	0.061	<0.001

ASC	*r*	0.270	0.168	0.610**	0.675**	1	0.576**	0.187	0.590**
	*P*	0.053	0.235	<0.001	<0.001		<0.001	0.184	<0.001

Caspase-1	*r*	0.112	0.066	0.428**	0.748**	0.576**	1	0.269	0.781**
	*P*	0.427	0.644	0.002	<0.001	<0.001		0.054	<0.001

IL-1β	*r*	0.533**	0.213	0.231	0.282	0.187	0.269	1	0.191
	*P*	<0.001	0.129	0.106	0.061	0.184	0.054		0.174

IL-18	*r*	0.290*	0.152	0.743**	0.812**	0.590**	0.781**	0.191	1
	*P*	0.037	0.282	<0.001	<0.001	<0.001	<0.001	0.174	

*Bivariate correlation analysis was carried out using the Pearson correlation method*.

*Statistically significant at P < 0.05.

**Statistically significant at P < 0.01.

### Enhanced Expression of Inflammasome Components in the Thyroid Follicular Epithelial Cell Line on Stimulation With IFN-γ and TNF-α

**Figure 5:**
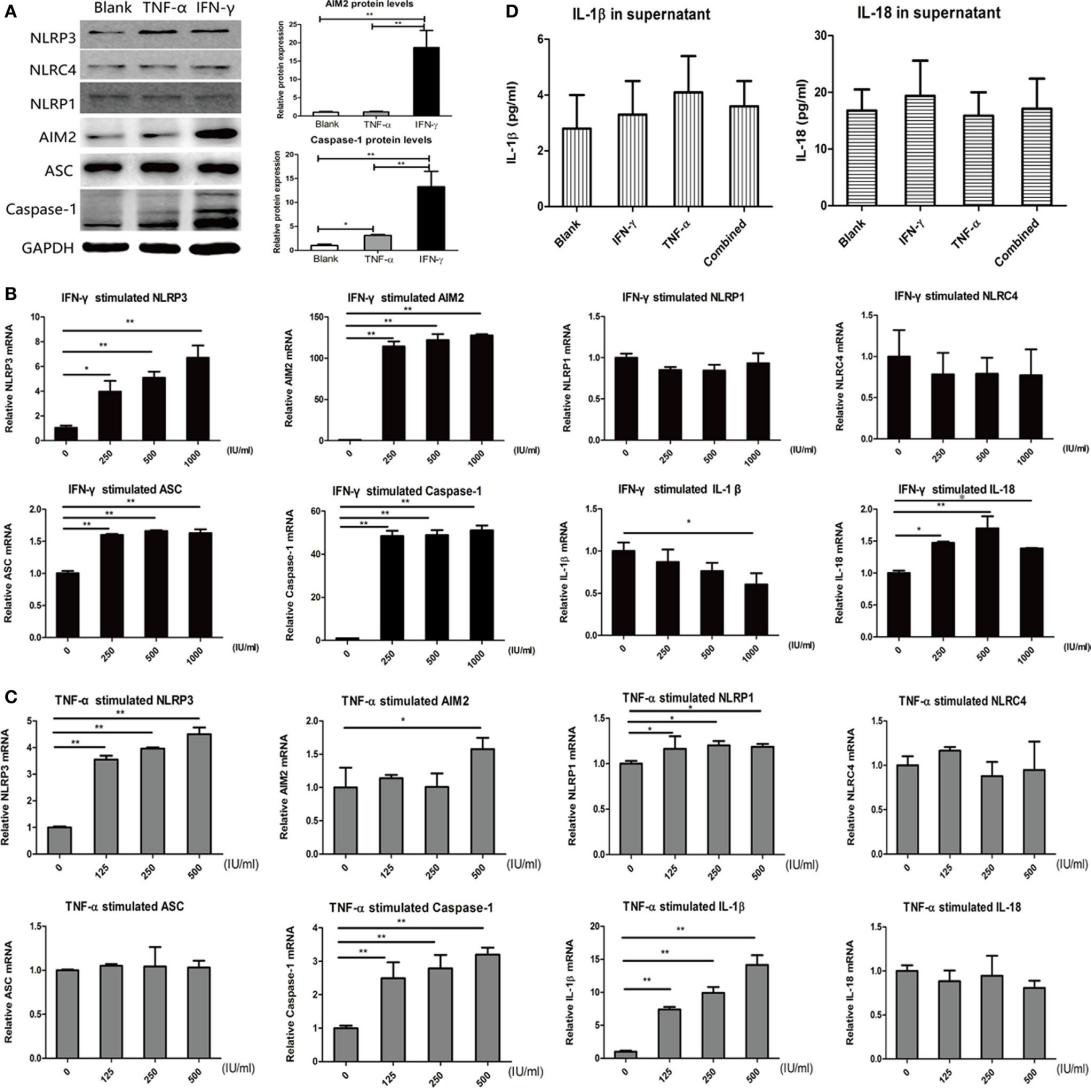
Increased expression of inflammasome components in thyroid cells on stimulation with IFN-γ and tumor necrosis factor-α (TNF-α). **(A)** Changes in NLRP3, absent in melanoma 2 (AIM2), NLRP1, NLRC4, ASC, and caspase-1 protein expression detected by western blot after incubation with 500 IU/ml IFN-γ or 500 IU/ml TNF-α for 24 h. Changes in NLRP3, AIM2, NLRP1, NLRC4, ASC, Caspase-1, IL-1β, and IL-18 mRNA expression detected by real-time PCR after incubation with **(B)** gradient IFN-γ (250, 500, and 1,000 IU/ml) for 24 h or **(C)** gradient TNF-α (125, 250, and 500 IU/ml) for 24 h. The relative mRNA and protein expression levels were corrected to those of untreated controls. **(D)** IL-18 and IL-1β levels in cell supernatant, as determined by enzyme-linked immunosorbent assay, after stimulation with 500 IU/ml IFN-γ, 500 IU/ml TNF-α or a combination of the two for 24 h. The columns represent the mean ± SD values. Data were obtained from three independent experiments. One-way ANOVA followed by Bonferroni correction was used for paired comparisons. *P < 0.05, **P < 0.01.

A human thyroid epithelial cell line was cultured to explore the effects of several cytokines on the expression of inflammasome components. TNF-α, IL-6, IFN-γ, and IL-17A, which have been reported to be present in AIT thyroid tissues and play important roles in the immunopathogenesis of AIT were used (27). As shown in Figure 5A, untreated thyroid cells express visible but low levels of the NLRP1, NLRP3, NLRC4, and AIM2 proteins. Incubation with gradient IFN-γ for 24 h significantly increased the mRNA levels of NLRP3, AIM2, ASC, caspase-1, and IL-18. In particular, compared with the blank controls, IFN-γ-stimulated remarkable mRNA and protein expression of AIM2 and caspase-1 (Figures 5A,B). As shown in Figure 5C, incubating cells with gradient TNF-α for 24 h significantly elevated the mRNA levels of NLRP3, caspase-1, and IL-1β, and slight but significant changes in NLRP1 expression were also observed. However, TNF-α stimulate AIM2 mRNA expression only at a high concentration. In contrast, stimulating thyroid cells with gradient IL-17A or IL-6 for 24 h did not influence the mRNA expression of inflammasome components (data not shown). The levels of both active IL-1β and active IL-18 in the supernatant, as determined by ELISA, did not differ between cells treated with IFN-γ only, TNF-α only, or IFN-γ combined with TNF-α (Figure 5D).

### Release of Active IL-18 on Activation of the AIM2 Inflammasome in Thyrocytes

**Figure 6:**
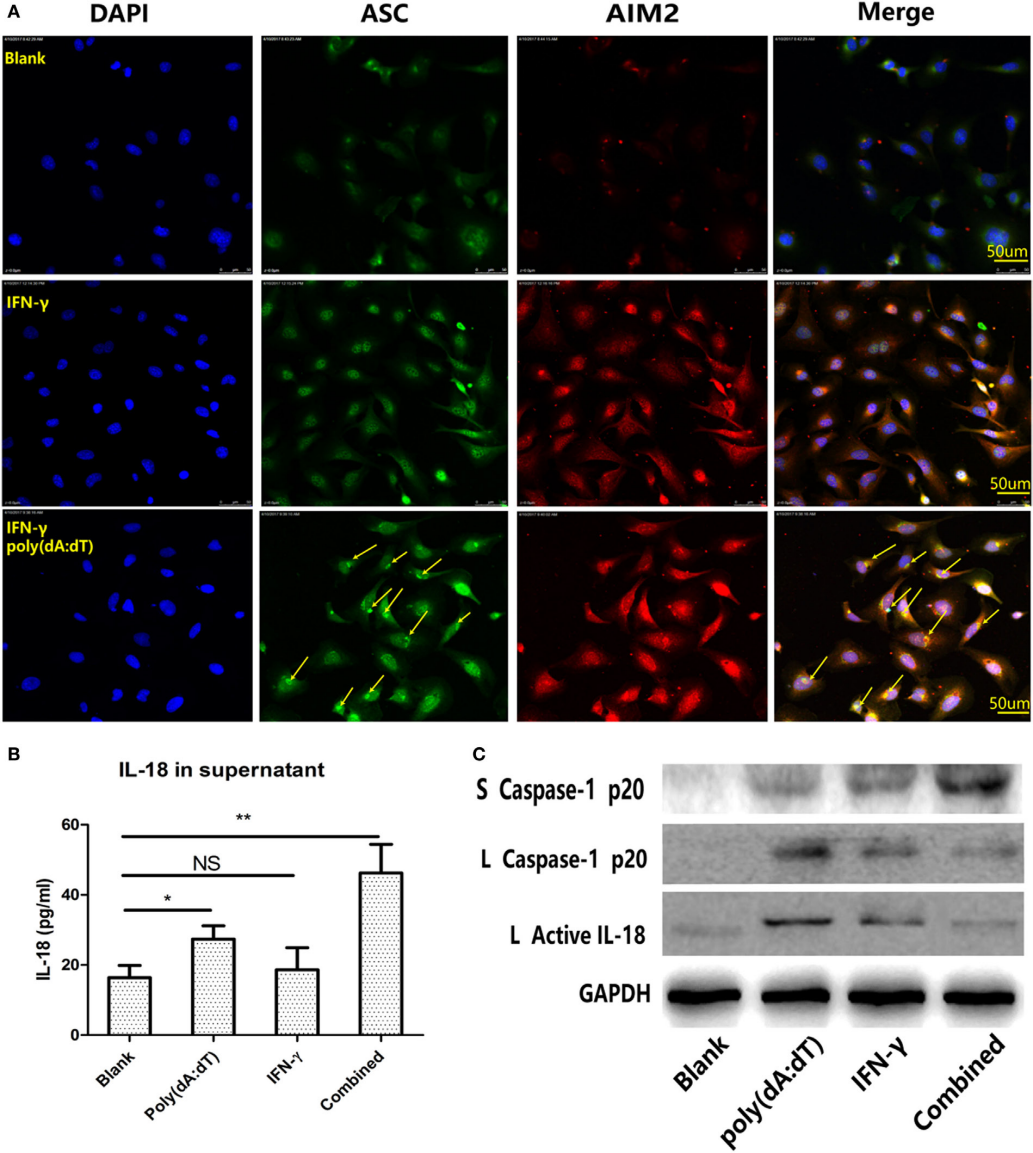
Promotion of poly(dA:dT)-induced activation of the absent in melanoma 2 (AIM2) inflammasome and release of active IL-18 in thyroid cells on IFN-γ stimulation. **(A)** Immunofluorescence analysis showed that ASC specks (green color, marked by arrows) were co-localized with AIM2 (red color) after the cells were stimulated with 1 µg/ml poly(dA:dT) for 12 h and pre-incubated with 250 IU/ml IFN-γ for 24 h (400× magnification, confocal laser-scanning microscopy). **(B)** IL-18 levels in cell supernatants determined by enzyme-linked immunosorbent assay after stimulation with poly(dA:dT) alone, IFN-γ alone, and both IFN-γ and poly(dA:dT). **(C)** Protein expression of caspase-1 p20 and active IL-18 in thyroid cells and supernatants after stimulation with poly(dA:dT) alone, IFN-γ alone, and both IFN-γ and poly(dA:dT). The columns represent the mean ± SD values. Data were obtained from three independent experiments. Statistical analysis was conducted by one-way ANOVA followed by Bonferroni correction for paired comparisons. *P < 0.05, **P < 0.01, NS, no significant difference; S, supernatants; L, lysates.

We observed a remarkable increase in AIM2 and caspase-1 mRNA expression (Figure 5B), as well as elevated protein expression of the AIM2 inflammasome components (Figure 5A) after stimulation with IFN-γ. Therefore, an AIM2 ligand, poly(dA:dT), was used to explore AIM2 inflammasome activation in thyrocytes. As shown in Figure 6A, macromolecular ASC specks (green color, marked by arrows) were observed to co-localize with AIM2 (red color) surrounding the nucleus after stimulating cells with poly(dA:dT) for 12 h after pre-incubation with IFN-γ for 24 h. These findings indicate that the assembly of inflammasomes may take place in thyroid cells. Elevated IL-18 levels in the supernatants were detected after poly(dA:dT) stimulation, and IFN-γ pre-incubation led to even higher IL-18 levels (Figure 6B). Increased caspase-1 p20 and active IL-18 proteins were detected in cell lysates stimulated with IFN-γ alone or poly(dA:dT) alone, but their level was decreased in the lysates of cells primed with IFN-γ and activated with poly(dA:dT); this may indicate the enhanced release of caspase-1 p20 and IL-18 into the supernatants of cells stimulated with IFN-γ combined with poly(dA: dT) (Figure 6C).

### Pyroptosis of Thyrocytes on Activation of the AIM2 Inflammasome

**Figure 7:**
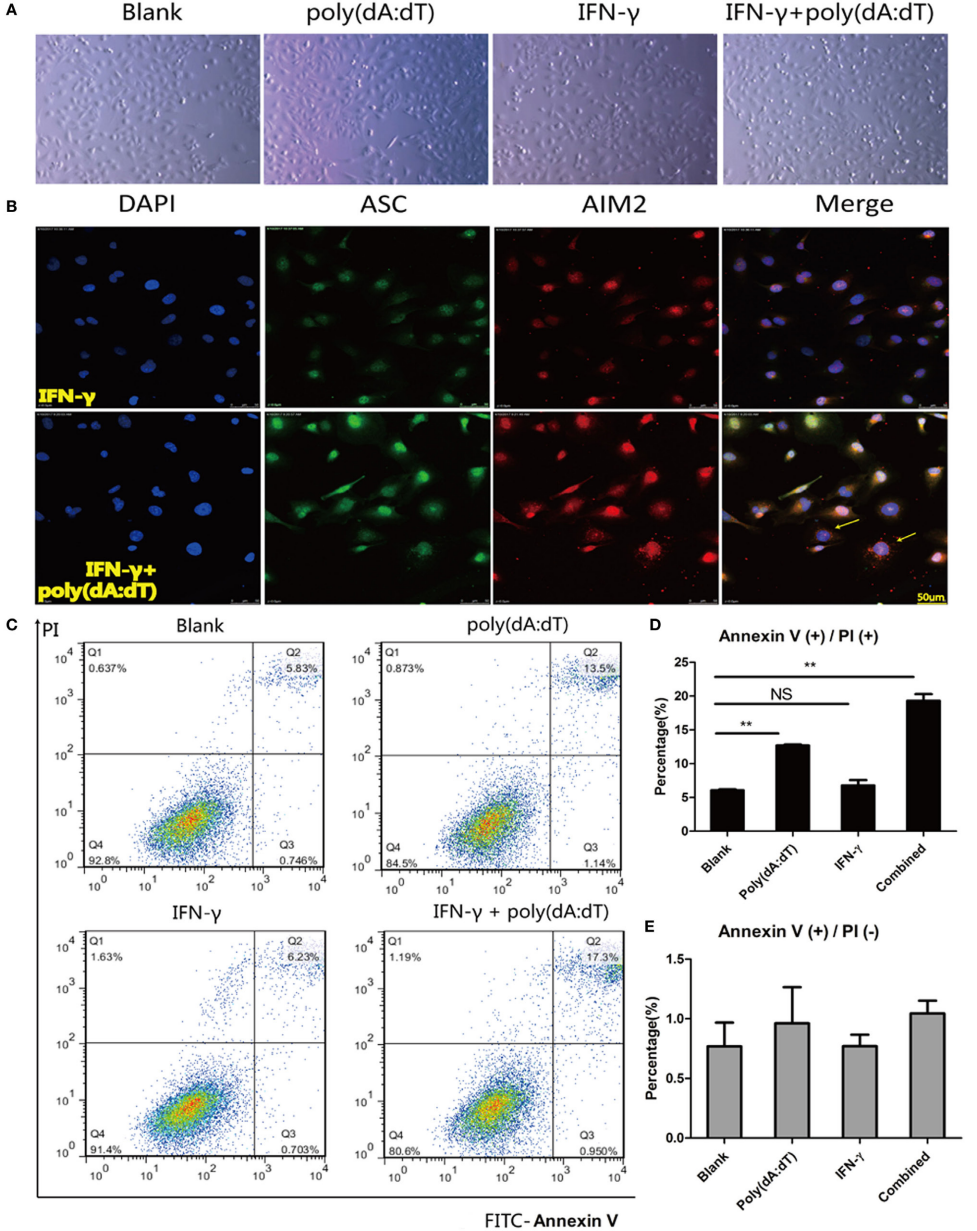

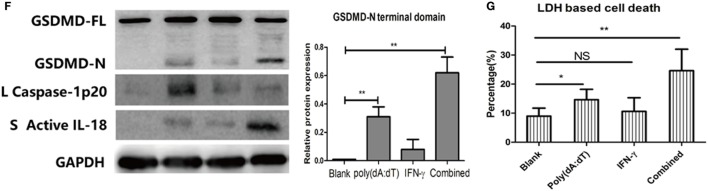
Promotion of poly(dA:dT)-induced thyroid cell pyroptosis on IFN-γ stimulation. Morphological changes in thyroid cells under a light microscope at 100× magnification **(A)** or under a confocal laser-scanning microscope at 400× magnification **(B)** after stimulation with 1 µg/ml poly(dA:dT) for 12 h after pre-incubation with 250 IU/ml IFN-γ for 24 h. **(C)** Representative flow cytometry images of FITC-Annexin V/propidium iodide (PI)-stained tissue after stimulation. Quantification of the percentage of Annexin V(+)/PI(+) and Annexin V(+)/PI(−) cells from flow cytometry findings **(D,E)**. **(F)** Protein expression of full-length gasdermin D and the N-terminal cleavage product in thyroid cells after stimulation with poly(dA:dT) alone, IFN-γ alone, and both IFN-γ and poly(dA:dT). Relative protein expression level was corrected to GAPDH expression. Caspase-1 p20 and active IL-18 expression indicates inflammasome activation. **(G)** Lactate dehydrogenase (LDH) levels in cells supernatants determined by absorptiometry after stimulation with poly(dA:dT) alone, IFN-γ alone, and both IFN-γ and poly(dA:dT). The columns represent the mean ± SD values. Data were obtained from three independent experiments. One-way ANOVA followed by Bonferroni correction was used for paired comparisons. *P < 0.05, **P < 0.01, NS, no significant difference; S, supernatants; L, lysates.

Pyroptosis is considered to be an important mechanism of active cytokine release resulting from changes in membrane permeability (28). Indeed, we observed more floating cells under a light microscope (Figure 7A) and puffy cells under the confocal microscope (Figure 7B) after IFN-γ pre-incubation and poly(dA:dT) activation, which indicates increased cell death. As shown in Figures 7C,D, flow cytometry revealed an elevated percentage of Annexin V(+)/PI(+) cells after poly(dA:dT) stimulation or poly(dA:dT) and IFN-γ stimulation compared with the blank controls. However, there was no significant difference in the percentage of Annexin V(+)/PI(−) cells among the studied groups (Figure 7E). Figure 7F shows increased N-terminal cleavage product (31 kDa) of gasdermin D in the only poly(dA:dT) group or poly(dA:dT) combined with IFN-γ group compared to the blank controls. Similarly, elevated LDH level was detected in cell supernatants after only poly(dA:dT) stimulation or poly(dA:dT) stimulation combined with pre-incubation with IFN-γ (Figure 7G).

## Discussion

In the current study, we have for the first time revealed the expression signature of several inflammasomes and the related cytokines in thyroid tissues from patients with AIT. Significantly increased mRNA and protein expression of NLRP1, NLRP3, NLRC4, AIM2, ASC, caspase-1, pro IL-1β, and pro IL-18 was observed in AIT patients compared to controls. Moreover, elevated expression of caspase-1 p20, active IL-1β, and active IL-18 was observed in thyroid tissue from AIT patients, which was indicative of enhanced activity of inflammasomes. These results together indicate that aberrant expression and activity of multiple inflammasomes are associated with the pathogenesis of human AIT. Consistent with our results, studies on other autoimmune disorders, such as multiple sclerosis (29), psoriasis (21), and lupus nephropathy (30, 31), have reported that overexpressed and hyper-activated inflammasome components in tissue cells play a role in autoimmunity. In addition, we found that the mRNA level of NLRP1 and ASC in thyroid tissues was correlated with the serum TPOAb and TgAb levels in the AIT group, which indicates that NLRP1/ASC could be potential biomarkers for AIT. Alkhateeb and colleagues have reported the correlation between single nucleotide polymorphisms of the NLRP1 gene and susceptibility to autoimmune thyroid disease (32); this may explain the changes in NLRP1 expression observed in the AIT patients in this study.

We investigated whether the elevated expression of inflammasomes in AIT thyroids was associated with the infiltration of mononuclear cells, thus immunohistochemistry on tissue sections was used to localize protein expression. Our result indicated that the expression of NLRP1, NLRP3, NLRC4, and AIM2 mainly localize in TFCs adjacent to lymphatic infiltration. Besides, since TFCs expressing inflammasome proteins were rarely seen in thyroid tissues without lymphocyte infiltration, we speculated that the pro-inflammatory cytokines secreted by infiltrating mononuclear cells and lymphocytes may be potential factors influencing the expression of these inflammasome components. Therefore, a human thyroid cell line was used to explore the effects of several AIT-related cytokines (27) on the expression of inflammasomes. Our results showed that IFN-γ and TNF-α had a priming but not activation effect on inflammasomes, because the mRNA and protien expression of multiple inflammasome components was significantly elevated in thyroid cells but without changes in active IL-1β or active IL-18 levels in the supernatant. Consistent with our results, previous studies have revealed that IFN-γ itself does not result in the release of active IL-18 (14, 21, 33). Similar to conclusions in human keratinocytes (21), IFN-γ-induced remarkable increase in AIM2 mRNA expression was also observed in our study. Thus, an AIM2 ligand, poly(dA:dT), was used to explore the biological effects of inflammasome activation in thyrocytes. We found an increased percentage of Annexin V(+)/PI(+) cells, elevated LDH levels in the supernatant, higher expression of the N-terminal domain of gasdermin D and elevated active IL-18 release after stimulation thyroid cells with poly(dA:dT); these findings indicate loss of cell membrane integrity and increased cell pyroptosis. Furthermore, IFN-γwas found to significantly strengthen poly(dA:dT)-induced release of active IL-18 and pyroptosis.

Based on the findings of the present study, two mechanisms by which inflammasomes contribute to the propagation of autoimmunity in AIT patients can be proposed. In the first mechanism, activation of inflammasomes in TFCs induces immune response by mediating cell pyroptosis and the subsequent release of bioactive cytokines. Enhanced expression of NLRP1, NLRP3, NLRC4, and AIM2 was observed mainly localized in TFCs that were near the areas of lymphocytic infiltration in thyroid tissue from AIT patients. Furthermore, cultured thyroid cells expressed visible proteins of these pattern recognition receptors and have the ability to respond to poly(dA:dT)—a typical DAMP. Similar to our conclusions, previous studies have demonstrated that human thyroid cells and the rat thyroid FRTL-5 cell line express a variety of functional toll-like receptors that recognize exogenous and endogenous threats and launch innate immune responses even in the absence of immune cells (6, 7). In the present study, we found increased pyroptosis of thyroid cells after poly(dA:dT) stimulation. Indeed, cell pyroptosis has been reported to cause inflammation and chemotaxis of immune cells through release of various DAMPs such as high-mobility group B1 protein and adenosine triphosphate (34, 35). Moreover, pyroptosis is considered to be an important mechanism of active cytokine release. In our study, elevated secretion of active IL-18 from the cultured thyroid cells was observed after stimulation with poly(dA:dT). Also known as IFN-γ-inducing factor, IL-18 evidently induces IFN-γ production and plays a major role in the Th1 response (36). In addition, previous studies on the thyroid tissues of patients with Hashimoto’s thyroiditis revealed upregulated IL-18 expression in TFCs located near infiltrating lymphocytes; this is similar to the distribution of inflammasome-related pattern recognition receptors in our study (14). Thus, we speculated that inflammasome-mediated maturation of pro IL-18 and release of active IL-18 from thyroid cells contribute to the extension of immune response in thyroid tissues of AIT. In the second mechanism, which forms a feedback loop with the first one, the pro-inflammatory cytokines secreted by infiltrated lymphocytes result in an increase in the expression of inflammasome components in thyroid cells and enhance DAMP-induced inflammasome activation. It is possible that different cytokines regulate the expression of different inflammasome components and may even work in synergy in this regard, since we observed that both TNF-α and IFN-γ upregulated NLRP3 mRNA expression in a concentration-dependent manner but TNF-α resulted in only a slight change in AIM2 expression only at a high concentration. Thus, we assume that different inflammasomes, alone or in combination, work in different types of inflammatory microenvironments.

Therefore, based on the present findings, we have proposed a feedback loop model in which inflammasomes lead to immune activation and their activity is simultaneously promoted by inflammation; this mechanism might play important roles in the cascade of follicular destruction and lymphatic infiltration in the thyroid of AIT patients. There are several limitations to our current study. First of all, we could not identify the accurate source of high inflammasome expression, because evidence is lacking to illustrate whether inflammasomes activation in thyrocytes or lymphatic infiltration/IFN-γproduction comes first. Second, the findings from the cross-sectional study could not illustrate the dynamic pathogenic role of different inflammasomes in different stages of AIT progression. Future investigations on AIT animal models at sequential time points of disease development might provide more specific conclusions. Besides, although IFN-γ and TNF-α have been considered play important roles in AIT pathogenesis, we did not verification the expression of these cytokines in AIT thyroid tissues in the current study. In addition, we only explored the effects of AIM2 inflammasome activation in the cultured thyroid cells using poly(dA:dT) alone or poly(dA:dT) combined with IFN-γ pre-incubation. Further investigations on other inflammasomes and multiple cytokines may help to explain their mutual interactions and their roles in cell pyroptosis and the release of active cytokines.

To conclude, we observed increased expression of several inflammasome components (NLRP1, NLRP3, NLRC4, AIM2, ASC, and caspase-1) and their downstream cytokines (IL-18 and IL-1β) in thyroid tissues from patients with AIT; these findings, along with the enhanced posttranslational modifications of caspase-1, IL-18, and IL-1β, are indicative of redundant activation of inflammasomes in thyroid tissue from AIT patients. The findings also indicate that poly(dA:dT) induces AIM2 inflammasome activation in thyroid cells, which increases cell pyroptosis and the release of active IL-18. Pro-inflammatory cytokines infiltrating the thyroid gland in AIT patients, such as TNFα and IFN-γ, promote the expression of multiple inflammasome components and enhance poly(dA:dT)-induced cell death and bioactive cytokine release. Based on the findings, an inflammasome-centered feedback loop model was proposed to explain AIT pathogenesis and its organ specificity. The identified inflammasome components could prove to be potential therapeutic targets and biomarkers of AIT in the future.

## Ethics Statement

All research procedures were approved by the Medical Ethics Committee of the First Hospital of China Medical University, and all participants provided their informed and written consent.

## Author Contributions

ZS, WT, and QG conceived and designed the research. QG, YH, YW, YLiu, TL, and HZ were responsible for subject recruitment and collection of specimens. QG, YLiu, and CF performed the experiments. ZS, WT, HG, YLi, and HZ contributed reagents, materials, instruments, and analysis tools. QG and ZS were responsible for data analysis and writing of the manuscript. All the authors listed have approved the final manuscript.

## Conflict of Interest Statement

The authors declare that the research was conducted in the absence of any commercial or financial relationships that could be construed as a potential conflict of interest.
